# Home-based therapy and its determinants for children with cerebral palsy, exploration of parents’ and physiotherapists’ perspective, a qualitative study, Ethiopia

**DOI:** 10.1371/journal.pone.0282328

**Published:** 2023-02-27

**Authors:** Zelalem Dessalegn Demeke, Yohannes Awoke Assefa, Yohannes Abich, Mulgeta Bayisa Chala

**Affiliations:** 1 Occupational Therapy department, School of Medicine, University of Gondar, Gondar, Ethiopia; 2 Physiotherapy Department, School of Medicine, University of Gondar, Gondar, Ethiopia; 3 Postdoctoral (OHT Impact) Fellow, Dalla Lana School of Public Health, University of Toronto, Toronto, Canada; Yenepoya University Yenepoya Physiotherapy College, INDIA

## Abstract

**Objective:**

This study aimed to explore the perceptions of parents and physiotherapists regarding home-based therapy programs for children with cerebral palsy and to understand the factors affecting adherence to home-based therapy programs.

**Materials and method:**

Thematic analysis method was used to identify, analyse and report findings. Twelve physiotherapists and five caregivers were purposively sampled and interviewed.

**Results:**

All transcripts were coded line by line, and the codes were then organized into categories for the development of descriptive themes and the generation of analytical themes. The data analysis followed the steps of the thematic analysis process. Seven themes emerged during the analysis: Why Home-Based Therapy? Ways of Teaching, Types of the therapy, Strategies of assessing adherence, Environmental factors, Attitude and knowledge; and Family participation. Physiotherapists use home-based therapy to prevent complications and improve functioning. They use various ways of teaching, such as explaining, demonstrating, and using pictures and videos. Physiotherapists consider several factors such as severity, age, and availability of resources before they decide the type of home therapy programs. However, parent’s participation was low; and strategies to monitor and evaluate adherence were also low. Low family support, limited recourse, lack of knowledge and poor attitude negatively affected adherence to home-based therapy.

**Conclusions:**

Our finding revealed that physiotherapists use quite limited methods of teaching, and do not properly monitor adherence of the home-based therapy. Additionally, family participation to select type of therapy and to set goal were low.

## Introduction

Cerebral palsy (CP) is the most common cause of disability among children disability with the childhood onset and lifelong implications [1, 2]. CP occurs in approximately 2–2.5 of 1000 live births globally [3]. In Africa, the prevalence varied greatly from country to country from approximately 2–10 per 1000 [4], but it is suggested to be higher than in western countries [4–6]. There is lack of a study on the prevalence of CP in the Ethiopian context. However, a study done in Uganda, a similar socio-economic status to Ethiopia showed the prevalence of CP were 2.9 per 1000 children [7].

CP is a group of non progressive abnormalities in the developing fetal or infant brain. Evidence indicates that there is variability of manifestations among children with CP, ranging from sensory, perception, cognition, communication, behavioral and musculoskeletal problems [8, 9]. Furthermore, children with the condition experience movement disorders, which further limits their functional capacity [2]. Rehabilitation has been suggested as the main management for children with CP, with the main goal of enhancing functional motor activity of a child [2].

Physiotherapists use various intervention strategies such as home based-therapy, and provision of assistive device to improve body function and social participation of children with cerebral palsy [10, 11]. For example, home-based constraint-induced movement therapy was found to be effective for children with hemiplegic cerebral palsy [12]. Another study by Bilde, Kliim-Due [13] also found that the home based internet therapy improved the motor ability, self-esteem, functionality and visual perception of children with cerebral palsy. Furthermore, home based program also was suggested as a cost-effective model of rehabilitation, particularly in resource limited settings where the primary cost of therapy is high and there is limited availability of rehabilitation centers and rehabilitation professionals [14].

Home based programs are lifelong activities and it is vital that children with CP adhere to the program to achieve the desired results [11, 15]. Developing functional skills in children with cerebral palsy requires repetitive practice in a variety of environments, including home settings, and that can not be achieved only by conventional, face-to-face therapy in rehabilitation centers [15].

Adherence is defined as the extent to which a client completes the active element of treatment effectively following advice and instructions [16] and comprises a wide variety of behaviors including entering into and continuing a treatment programme, attending therapy appointments and performing home-based exercises [17].

Home based therapy needs to follow holistic approach that take the child and their family’s preferences into an account [18]. Children with cerebral palsy depend on their parents or caregivers to perform their home-based therapy program. Since the perspectives of the child’s parent or caregiver play central role in the adoption and use of home-based program [18], it is recommended that the program is family-centred approach [19]. Families of children with CP are regarded as experts on their child’s needs [20–23]. Hence, it is suggested that rehabilitation professionals and the families of children with CP collaboratively make decisions, design and implement specific home-based program to improve the child’s function [21, 22, 24–27].

A few available studies on parent-implemented intervention for children with CP in low and middle income countries indicated that home based therapy program found to improve child’s bimanual hand and chewing function [28]

A number of factors determine the success of home-based program [18, 29, 30]. According to Peplow and Carpenter [18], child’s motivation, time management and perception of physiotherapists regarding the home based exercises determines adherence to the home based therapy. Moreover, the characteristics of the home-based therapy and the way physiotherapists explain and demonstrate the home exercises also affects the uptake and adherence to the program [29]. Demonstrating the exercises while the parents are watching; asking the parents to demonstrate the exercises at least once per month; observing exercise practice while providing feedback and making subsequent corrections; giving written instructions and explanations in a way that is accurate, and simple to understand found to increase the adherence [29]. Conversely, when parents perceive the home therapy is too complex and causing adverse effect; they tend not to adhere to the therapy [29]. In addition to the above factors, environmental factors such as emotional and physical support from family members, provision of equipment, provision of an exercise logbook; and personal factors such as motivation, autonomy, and time management are found to significantly affect home exercise adherence [31].

Presently, there are several gaps in the literature related to CP related home-based exercise program in Ethiopia. Considering the socio-economic, cultural, and literacy differences between high income countries and middle-income countries such as Ethiopia, we anticipate the perception of home-based exercise program for children with CP could be different. Hence, we believe that it is imperative to conduct this study in our setting.

Moreover, in Ethiopia, rehabilitation centers are located only in major cities and inaccessible for most families [32]. Hence, home based therapy programs appear to be an efficient and cost-effective solution for such settings.

Understanding barriers and facilitators to adherence to home therapy program for children with CP from physiotherapists and parents’ perspective will help in designing relevant strategies to encourage adherence. The aim of this study is two-fold: a) to explore the perceptions of parents and physiotherapists about home therapy programs for children with CP, and b) to understand the factors related to adherence to home therapy programs from physiotherapists and parents’ perspective.

### Research questions

1. What are the perceptions of physiotherapists towards home-based therapy programs for children with cp?
2. What are the perceptions of parents toward home-based therapy programs for children with CP?
3. What are the factors that affect adherence to home therapy programs from physiotherapy and parents’ perspective?

## Method

### Study design

This study used a thematic analysis method to identify, analyse and report findings. Thematic analysis is a preferred qualitative research method to identify, analyse, organize, describe and report themes within data; which also yields a rich an detailed output [33, 34].

### Participants

A purposive sampling strategy was used to recruit 12 Physiotherapists with a wide range of characteristics, including age, education level, experience, and place of work. Physiotherapists who have at least two years of experience in physiotherapy clinical service and who worked with children with CP were selected. We set two years of experience because we believe that since PTs in most settings work in rotation, within two years, they will have a probability of working in pediatric clinics. The physiotherapists were recruited from pediatric rehabilitation centers at the University of Gondar hospital in Gondar and Addis Pediatric Rehabilitation Center in Addis Ababa. Five parents who have at least one child with cerebral palsy diagnosis and those who have been given a home-based program by a physiotherapist were also included in the study. These participants were recruited from Addis Pediatric Rehabilitation Center, Addis Ababa. Informed consents were obtained from all participants before the start of each interview. The sample size was determined based on data saturation.

### Procedure

Individual, face-to-face, and semi-structured interviews were the method used to collect data. Separate semi-structured interview guides were developed for both parents and therapists by the authors. The guides contain open-ended questions designed to explore the perception and experience of physiotherapists and parents of children with CP regarding home-based programs. The questions were first sent to one physiotherapist, who is working as a clinician and lecturer at the university, prior to the interview for validation. The information obtained from the physiotherapist’s perspective was triangulated with the parents of children with cerebral palsy. Several prompts were also asked when there was a need to gain the participants’ experiences in depth. Interviews were conducted in Amharic which is the official language of the Federal Democratic Republic of Ethiopia [35]. The first author (ZDD) conducted all the interviews. Data were collected in a quiet place at the rehabilitation centers. Field notes were also taken during and immediately after each interview. All interviews were face-to-face and lasted between 40 to 60 minutes. All interviews were audio recorded and transcribed verbatim in the language of the interview. Participants were informed about the purpose of the study and their right to withdraw from the study at any point of time. They were also assured that their responses and identities would remain confidential and not revealed in research reports.

### Data analysis

The interviews were transcribed verbatim in Amharic, and transcripts were compared to the recordings for accuracy. Transcripts and field notes were analyzed using an inductive thematic analysis approach [33]. There were six steps to the analytic process: 1) familiarization with the data, 2 (generating initial codes, 3) searching for themes, 4) reviewing themes, 5) defining and naming themes, and 6) writing and discussing the findings. The analysis started with data familiarization by listening to the audio recordings, reading, and rereading the transcripts [33].

The transcribed documents were imported to NVivo 12 plus software for the purpose of coding and thematization. All the transcripts were coded line by line, and these codes grouped into categories. The first and second author independently coded three transcript to develop a coding tree. The third author was consulted when consensus was not reached between the first and the second authors. Data analyses were performed by all authors, and all codes and categories were confirmed with the research team during periodic meetings.

Member check, peer check, and Audit trial were used for the sake of trustworthiness. For member check, we gave the written copy of the finding to some participants to confirm whether the data fit to what they said or not. Also, we asked colleagues who have experience on qualitative studies to review our transcripts (peer check). Audit trail was also kept by two investigators who kept records of raw data, field notes, and transcripts.

### Ethical approval and consent to participate

This study was approved by the Institutional Review Committee of the College of Medicine and Health Sciences, the University of Gondar, Ethiopia with the reference number V/P/RCS/05/496/2020. Official letters from the administrators of the University of Gondar were obtained before contacting the study participant. The study’s purposes and importance have been clarified to each participant. They were informed that participation in the study was voluntary.

## Results

### Sociodemographic factors

Seventeen participants, twelve physiotherapists and five parents participated in the study. Eight of the physiotherapists were with BSc degree in physiotherapy and four had MSc degree in physiotherapy. All five parents were female, mothers of children with CP. Details of the sociodemographic of the participants is illustrated in the table below (Tables 1 and 2).

**Table 1:** Sociodemographic factors of caregivers of CP children.

ID	Caregiver relationship	Caregiver sex	Caregiver age	Caregiver occupation	Household income (in ETB)	Child age	Child sex	Physiotherapy service follow up
Caregiver 1	Mother	Female	34	Home maker	3,000	2yrs and 7m	Male	3months
Caregiver 2	Mother	Female	37	Government Employee	9,000	4yrs and 2m	Male	1year
Caregiver 3	Mother	Female	25	Government Employee	7,500	1yr and 9m	Female	4months
Caregiver 4	Mother	Female	29	Home maker	5,000	1yr and 3m	Female	1year
Caregiver 5	Mother	Female	30	Home maker	4,500	3yrs	Male	2yrs and 6months

**Table 2:** Sociodemographic factors of physiotherapist.

ID	Education level	Age	place of work	Sex	Experience working with CP child (in years)	Training regarding CP
PT 1	BSc	25	Addis Ababa	Male	1year and 5months	No
PT 2	BSc	27	Addis Ababa	Male	3years	No
PT 3	BSc	25	Addis Ababa	Female	2years	No
PT 4	MSc	30	Gondar	Male	4 years	No
PT 5	MSc	28	Gondar	Male	5 years	No
PT 6	BSc	24	Addis Ababa	Female	2 years	No
PT 7	BSc	24	Gondar	Male	1year and 6months	No
PT 8	BSc	26	Gondar	Male	3 years	No
PT 9	MSc	29	Gondar	Male	4 years	No
PT 10	MSc	30	Addis Ababa	Male	5 years	No
PT 11	BSc	24	Addis Ababa	Female	2 years	No
PT 12	BSc	25	Gondar	Female	2 years	No

### 1. Why home-based therapy?

Almost all PTs responded that the therapy provided at the clinic is insufficient to achieve the desired results, so they prescribe home-based therapy to achieve the outcome they intend to see.

“*They come here once in a week*, *or once in a month which is not enough so we give them the exercises to do at their home more frequently*,*” said the PT participant “I see the improvement sooner when I add home exercises on the regular therapy*, *than the regular clinic-based therapy*” added the PT.

Others recommend the home-based therapy to prevent further complications such as contracture and to reduce further disability. For instance, one PT described, “*this [the home exercise] helps to decrease the spasticity which prevents future risk of contracture*.” Moreover, PTs mentioned the importance of home-based therapy on improving digestion, balance and stimulating muscle activity.

Similarly, others stated the home-based therapy helps to improve the functionality of the unaffected part. One PT used an example of one of her clients’ experiences “*I give him the exercises so he can practice on his unaffected part and that helps him to improve his functionality*.”

The PTs also suggested that home-based therapy program is beneficial for patients living in rural areas. As explained by the participants, these patients often face several hurdles including lack of transportation to come to cities to have a clinic/hospital-based therapy. Hence, PTs use home-based therapy to substitute the institution-based therapy.

Despite that PTs hold positive perception towards the importance of home-based therapy for children with CP, none of them ever take special training to work with children with CP. They, however, stressed on the importance of trainings, specific to cerebral palsy, which will put them at the better place to be able to provide the best possible care for children.

### 2. Diverse ways of teaching

All interviewed physiotherapists strongly asserted the importance of using various methods of teaching the home-based therapy exercises to improve adherence of the program. Hence, they use various methods of teaching. The findings show that all physiotherapists demonstrate and explain the therapeutic exercises on the child and then ask caregivers to show back what they have learned. Three of the participants mentioned that they use videos and pictures to teach the exercises to the parents. One of the PT participants let the parent to record video of them while demonstrating the home exercise, so caregivers can later refer to it.

The importance of using few and simple exercises that can be done using the locally available equipment were also raised in the interviews. One physiotherapist stated “… *I always try to limit the exercises up to three at a time*, *so they don’t forget the steps* …” By the same token, physiotherapist’s have also tried to encourage the caregivers to use locally available materials for the home exercises. For instance, one physiotherapist stated “*I encourage them [parents] to use blankets to replace a CP chair when they cannot afford to buy a CP chair*…” Furthermore, the participants also emphasized the importance of using communicating patients using less complex terms and metaphors to explain the home exercise. For example, one physiotherapist stated, “…. *Do the home exercises three times a day as if you eat three times a day*…”

Similarly, all physiotherapist participants also check back with the caregivers whether they understood what to do at home “*we ask them to explain back to us about what they have been shown…*. *we also ask them to demonstrate back*.” However, there is a limitation in monitoring the adherence of the home-based therapy exercises.

### 3. Limited strategies of assessing adherence

Although the physiotherapists stated the importance of monitoring the home-based therapy adherence, they use only a handful of strategies to evaluate the level of adherence to the prescribed home-based therapy. Only one of the PT participants use phone calls to monitor. The majority of PTs rely on the feedback from the child’s caregiver whether or not they are following the home exercises. The findings also show that a few PTs ask caregivers to demonstrate the home exercises to PTs during their clinic appointment time to evaluate if they are following the exercises correctly. One PT said, “*There is no other way but just asking the mom if they are doing {the home therapy} or not*.”

Also, another PT mentioned that he develops his own follow-up tool and uses that to evaluate adherence. Whereas others assess the improvement to check if the home exercise has been done or not. For example, one PT stated “…. *For a child whom his mom has done the home exercises*, *his contracture will lessen*…” However, the improvement could be resulted from different factors than the adhered home based therapy exercise.

Despite the limited strategies PTs use to assess adherence, they indicated the importance of using different methods following up, such as using phone calls and using community-based rehabilitation workers to visit children at their homes.

### 4. Not all CP children get the same type of home-based therapy

The PTs indicated several factors to consider before prescribing home based therapy to children with CP. This includes severity of the disability, age of the children, and availability of resources. PTs assess the level of disability and set their own goal on how much progress they want to see. Then, they prescribe home exercises in order to attain those goals. However, all of the PT interviewees mentioned that neither parents nor children participate in goal setting process. This does not align with the current best approach, which is family-centered therapy.

The goals and the type of the home exercises are based on the severity of the disability. The severity is sometimes decided by the level of GMFCS or by just based on what the therapists believe. For example, one PT stated, *“if the child has contracture*, *we [I] will give him [CP child] a passive range of motion exercise*”

By the same vein, PTs also consider the age of the child and their developmental milestones before they recommend they type of home-based exercises for children with CP. One participant said: “… *for example*, *if he is at school age*, *I would give him exercise that relates with his school…*”

The Physiotherapists also take into account of environmental factors such as availability of materials (and ability to buy), residence and the child’s home environment to decide on the type and nature of the home-based therapy programs.

### 5. Environmental factors

In this theme physiotherapists and parents of children with cerebral palsy discussed about the economic, environmental and resource limitation factors to adhere to the home-based therapy program. Additionally, parents who have additional competing roles, including taking care of other children and other member of the family limit their involvement in the home-based therapy exercises. This eventually has negative effect on completing the home exercise as it is prescribed. For instance, one PT said “the *main reason that parents told me not to carryout the home exercise is poverty*, *they prefer to work in income generating activities that can feed their family than taking care of their child with CP*”

Parents’ experience also proves the physiotherapists’ perception of environmental barriers for adherence to the prescribed home-based therapy exercises. As discussed during the interviews, parents are usually occupied by taking care of other children and on multiple house chores, which decreases their motivation and energy to engage in home-based therapy programs. For example, one mother stated: “I have to work all day to feed my children, then I have to take care of his [child with CP] siblings, I get tired….it is tiring…”

Furthermore, the mothers discussed the lack of support from other family members on providing care for their CP child. One participant said, “…. *I am the only person who is supposed to take care of him [her child with CP]*”.

Finally, the participants discussed a narrow home space and limited therapy equipment as a barrier to providing home-based exercises for children with CP. PT participants explained that they encourage parents with CP children to adapt and use locally available materials to provide home based exercises to the child. Nevertheless, caregivers mentioned that they struggle to get and use materials that can support the home exercise. For instance, one PT mentioned “*materials such as playing toys to be used by two hands and CP chair would improve the adherence of home therapy*.”

### 6. Lack of awareness and poor attitude affects adherence

The findings from the interview indicated the presence of several personal factors such as lack of awareness about CP and negative attitude towards exercises, that affect their motivation to engage in exercises, which results in poor adherence to home-based therapy. As discussed by the participants, lack of parent’s awareness about the cause of CP was the main reason why parents are not engaging their children in home-based therapy. For example, a PT said, “*Most parents think the disability comes from curse or God*, *and they usually prefer to go to spiritual places than keep doing the exercise*” said one PT.

Additionally, parents believe that only drugs, not “exercise,” can cure their child’s condition. This eventually decreases their motivation to adhere to the home-based therapy. Similarly, the inability to see changes in the child’s condition in short period of time discourages parents from adhering to home-based therapy. This could be due to the lack of knowledge about the condition. When parents cannot see progress in short period of time, in addition with other factors such as lack of support, mothers usually lose their motivation, and hope, to adhere to the exercise.

“*The main reason is awareness*” said the PT participant “*parents usually are not aware about the importance of physio [exercises]*”. As a result, even though parents keep doing the exercises they do not do as it is prescribed, they just do it so they can say they do the exercises.

### 7. Family perception and participation

In this study, we intended to understand the family members’ perception about the importance of the home-based therapy program. Despite the negative attitude explained above, the mothers who participated in this study discussed the importance of increasing the frequency of home-based exercise sessions to yield a positive outcome in their child’s condition. One mother stated, “*I come here [the hospital] once a month*, *which is not enough for my child*, *but I can do the home exercises daily*, *which is as required*.”

Similarly, we assessed the level of family participation in prescribing the home based therapy. The in-depth interview revealed that the level of family participation was low in the process of choosing and prescribing the home-based therapy exercises. The PTs solely set goals, choose the exercises and tell families what to do. All PT participants considered participating family is only letting families know the plan and answering the questions families might have.

However, PT participants ascertain the importance of including parents in the process of choosing and prescribing home exercise and showing the changes to parents. This will motivate parents to keep doing the home exercises as they will be optimistic about the future of their children. Furthermore, PTs also indicated the importance of showing the changes for the parents, to motivate parents to adhere to home exercise therapy. Also, including the expectation of parents’ is indicated as a solution to improve adherence.

## Discussion

This is the first qualitative study that has explored home based therapy program for children with CP from the perspective of physiotherapists and family of children with CP in Ethiopia. As discussed in the results section, our in-depth interviews have shown that PTs use various methods of teaching of home-based therapy. The methods discussed by the PTs such as the use of demonstration, illustrating the nature and steps to the exercises of exercises using videos and pictures, is similar to the methods used elsewhere [29, 31]. Lillo-Navarro, Medina-Mirapeix [29] and Taylor, Dodd [30] discussed that demonstrating the exercise, asking parents to demonstrate back at least once per month, provide written instructions and exercise logbook helps to improve adherence. The authors also found that providing an exercise logbook that contain detailed information about the exercise, such as what to do, how frequent to do highly increases adherence to the home-based therapy. Similarly, Jeglinsky, Autti-Rämö [22], in their study, revealed that providing written information about the child’s condition and the therapy improves the outcome of children with CP. However, our finding reveled that only few PTs provide pictures and videos of the exercises, and none provide exercise logbook. PTs in low resource setting such as Ethiopia, can prepare and use exercise logbook with low cost to improve adherence.

According to our finding, the main strategy PTs use to assess the adherence to the home-based therapy is by subjectively asking the caregivers whether they are doing the exercises or not. Others, objectively assessed by asking parents to demonstrate. And only one-use assessment tool to measure adherence. However [29] stressed the importance of monitoring the home exercise program by PTs’ using both subjective and informal methods. PTs in the setting can develop or adopt adherence checking tool to objectively monitor the outcome and evaluate adherence. In addition, PTs can use phone call, and use the community rehabilitation workers to support and check adherence.

Typically, Ethiopian women engage in multiple indoor and outdoor activities. They are usually the one who is taking care of children, home chores and also work on income generating activities to support their family. We found similar in our study, that mothers are busy doing the aforementioned activities decreases their adherence to complete the home-based therapy exercises. On top of that, it was evident in our study that they receive poor family support which adds another layer of barrier to mothers in facilitating home-based exercise programs. This finding is not unique to our context. Previous studies conducted by Lillo-Navarro, Medina-Mirapeix [29], Taylor, Dodd [31] indicated that time management of care givers, and availability of support in the family greatly affects the success of adherence. Lillo-Navarro, Medina-Mirapeix [29] discussed that one of the main reasons that hinders parents from adhering to the home exercises is that home exercises are time taking and restrict their time for recreation or relationships with their children. PTs in Ethiopia can work closely with parents on time management so parents can find time in a day to complete the home-based therapy. Furthermore, PTs can work with family members to improve participation of all family members on taking care of children with CP.

Moreover, our finding reveled that, resource limitations to get materials that support the home exercise and narrow home space were identified as barriers to adhere to home-based therapy. Our findings are in line with the study done by Taylor, Dodd [31] and Peplow and Carpenter [18]. Taylor, Dodd [31] indicated that availability of suitable exercise equipment influences adherence. By the same token, Peplow and Carpenter [18] identified in their study that, lack of appropriate equipment to support home exercise decreases adherence of parents and children to adhere to the prescribed home exercises. PTs in Ethiopia use their problem-solving skill to help parents to use locally available materials for the home based therapy.

Despite the crucial role that family plays in the therapy process of children with CP [18, 20–22, 25–27], the current study revealed that the actual family participation is low in the process of home based therapy. Letting families know what to do, asking them if they have question and addressing the questions they might have been solely considered by PTs as family participation. Family centered therapy, however, goes beyond dictating families what to do, and answering questions they may have; but seeing the parents as experts on their child’s condition and need, and collaborate with them in decision making and goal setting [20–23]. Taylor, Dodd [31] also revealed that adherence to home-based therapy improved when participant’s decision role in the process of therapy is increased. Novak and Cusick [36] also discussed in their work the five phase of family participation in home programmes for children with CP, namely establish a collaborative role with the caregiver, establish mutually agreed goals, select therapeutic activities, implement selected activities, and finally evaluating the program.

Finally, our findings revealed several personal factors such as poor knowledge about the condition, lack of motivation, and poor caregivers’ attitude towards CP and home-based therapy decreased the adherence of home-based therapy. A similar study conducted in South Africa revealed that increased public awareness about the cause of disability facilitates the parents care towards their children with CP [30]. Likewise, a study done in Ghana revealed that parents who have adequate knowledge about CP better cope with the demands of taking care of their children with CP [37]. Moreover, child’s and parent’s motivation to do the home exercises were also indicated as a factor for adherence [18, 31]. Hence, in Ethiopia, PTs, other health workers, government bodies and the media can work towards increasing general population’s and parents’ knowledge regarding CP. Moreover, PTs can use different strategies to improve parents’ motivation to adhere to the home exercise. For example, PTs can use positive reinforcements, and show the progress of the child to parents.

Limitation of this study could be the implications might not be generalized to other settings.

## Conclusions

The aim of this study was to explore the perception of parents and physiotherapists regarding home-based therapy programs for children with CP and to explore the factors that barriers or facilitates adherence to home-based therapy programs from physiotherapists and parents’ perspective. Our finding revealed that physiotherapists use quite limited methods of teaching, and do not properly monitor and evaluate the adherence of the home-based therapy. Additionally, family participation in the process of selecting the activities and setting goal found was low. The aforementioned factors and poor support, lack of adequate knowledge about the condition and busy schedule of parents’ barriers parents from adhering to the home-based therapy.
