# Correction: Home-based therapy and its determinants for children with cerebral palsy, exploration of parents’ and physiotherapists’ perspective, a qualitative study, Ethiopia

**DOI:** 10.1371/journal.pone.0315114

**Published:** 2024-12-02

**Authors:** Zelalem Dessalegn Demeke, Yohannes Awoke Assefa, Yohannes Abich, Mulugeta Bayisa Chala

The fourth author’s name is spelled incorrectly. The correct name is: Mulugeta Bayisa Chala.

There is an error in affiliation 3 for author Mulugeta Bayisa Chala. The correct affiliation 3 is: Postdoctoral Fellow, Institute of Health Policy, Management & Evaluation, Dalla Lana School of Public Health, University of Toronto, Canada.
